# β-Casein A1 and A2 Genetic Variants and β-Casomorphin-7 in Raw Milk and Processed Milk Products

**DOI:** 10.3390/ijms26178612

**Published:** 2025-09-04

**Authors:** Stanisław Kamiński, Anna Cieślińska

**Affiliations:** 1 Department of Animal Genetics, University of Warmia and Mazury in Olsztyn, 10-719 Olsztyn, Poland; stachel@uwm.edu.pl; 2 Department of Biochemistry, Faculty of Biology and Biotechnology, University of Warmia and Mazury in Olsztyn, 10-719 Olsztyn, Poland

**Keywords:** milk products, β-casein, genetic variant, A1 and A2

## Abstract

The A1 and A2 variants of bovine β-casein (CSN2) have gained attention in the dairy industry due to potential health effects. The A1 variant, prevalent in Holstein-Friesian cattle, is a major source of β-casomorphin-7 (BCM-7)—an opioid-like peptide released during digestion and associated with lower digestive comfort. In this study, β-casein A1 and A2 variants and BCM-7 levels were quantified in raw milk and three commonly consumed dairy products (pasteurized milk, UHT milk, and milk powder) using ELISA. The samples came from dairy plants within a single operating zone. The A1 variant was significantly more frequent (13.69–22.41 ng/mL) than the A2 variant (8.10–12.60 ng/mL), although the local Holstein cattle population had a higher frequency of the A2 allele (63%) than A1 (37%). This discrepancy could be due to a more efficient expression of the A1 allele in cows with heterozygous or A1A1 genotypes. BCM-7 levels were low and did not vary significantly with *CSN2* genotype or processing method. These results provide new insights into the composition of dairy products and contribute to the ongoing debate on the health implications and consumer acceptance of milk with the A1 β-casein variant.

## 1. Introduction

Since ancient times, cow’s milk and other dairy products have been regarded as almost wholesome foods that provide children and adults with many important nutrients [1]. Holstein-Friesian cattle are the most widespread breed in the world and produce milk as a raw material for a variety of dairy products [2]. Among milk proteins, caseins are the most important components of milk and play a crucial role in milk processing [3]. It is known that β-casein (CSN2), which is encoded by the *CSN2* gene, accounts for up to 5% of the total caseins in bovine milk. At least 15 protein variants have been identified and characterized in cattle (A1, A2, A3, B, C, D, E, F, G, H1, H2, I, J, K, and L), of which A1 and A2 are the most common. I is less common, and B, A3, and C are rare [4,5,6]. One of the most extensively studied variants is the nucleotide substitution X14711:g.8101C>A, which causes an amino acid change from proline to histidine at position 67 [7].

The presence of cytosine at this position is consistently associated with the appearance of β-casomorphin-7 (BCM-7), a bioactive peptide. BCM-7 may exhibit a range of immunological activities and has been implicated as a potential risk factor for insulin-dependent diabetes, atherosclerosis, and ischemic heart disease. On the other hand, the beneficial A2 variant has shown some negative effects on the properties of milk in cheese production. For example, the A2 variant has been associated with slightly prolonged rennet clotting time and reduced curd firmness—two properties that are of great importance in cheese production [8]. Other studies have also reported that milk with the A2 variant has several disadvantages in this process. Furthermore, it was found that a higher amount of CSN2-encoded β-casein in milk significantly affects cheese production, although no significant differences were observed after seven days of ripening. Consequently, nutrient recovery showed a more efficient process when *CSN2* A2 variant was present [9,10].

In addition to the A1 and A2 variants, the β-casein B variant also deserves attention due to its technological significance. This allele has been associated with higher proportions of β-casein in the protein fraction, smaller micelle size, and favorable effects on milk coagulation, including shorter rennet coagulation times, faster curd firming, and generally improved cheesemaking properties [11,12,13,14,15]. However, some studies suggest that the B allele may slightly worsen certain coagulation parameters, and it is often considered together with the A1 group of alleles.

**Figure 1:**
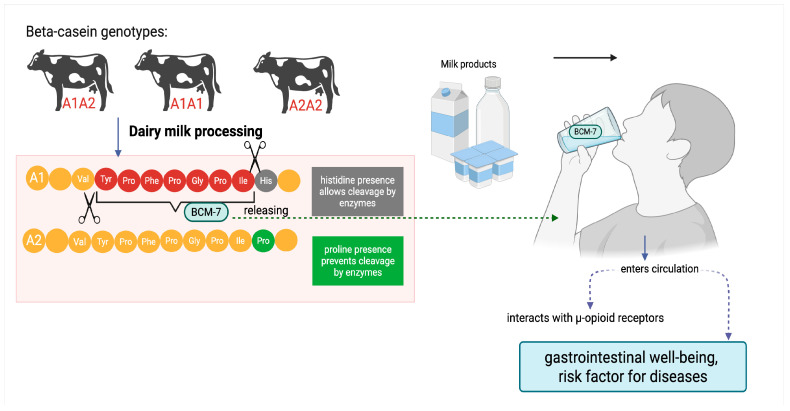
Proposed mechanism of β-casomorphin-7 (BCM-7) release from β-casein A1 variant during gastrointestinal digestion. Variant A2 does not generate BCM-7.

The A2 allele is now described as the ancestral variant, while A1 variant is noted as a derived allele resulting from a point mutation in *Bos taurus* breeds, with the highest frequency in modern Holstein-Friesian dairy cattle [16]. For more than 25 years, there has been an ongoing scientific debate about the potentially harmful effects of the A1 variant on human health. Numerous studies suggest that the digestion of A1 cow’s milk leads to the release of BCM-7, an opioid peptide associated with gastrointestinal discomfort and an increased risk of various diseases [17,18,19] (Figure 1). Based on these findings, A2 dairy products (which do not contain the A1 variant and only traces of BCM-7) have been marketed in many countries as a high-quality alternative. The global market for A2 dairy products is expected to reach USD 3.7 billion by 2027 [20].

The milk processed in the dairy plant is bulk milk because it comes from cows that have one of the most common *CSN2* genotypes (A1A1, A1A2, or A2A2). The content of A1 and A2 variants in bulk milk should therefore reflect the average frequency of these variants in the cow population held in the dairy’s operational areas. These frequencies have already been estimated in many countries, e.g., France [21], Italy [22] and Poland [23]. However, our last report showed that the A1 variant dominates over the A2 variant [16], which might influence the actual content of β-casein variants A1 and A2 in dairy products.

Genetic studies on β-casein variants in dairy cows, the content of the A1 variant in milk, and the concentration of β-casomorphin-7 have usually been conducted in separate studies [6,24,25], which may lead to uncontrolled variability due to environmental factors. In the present study, we aimed to minimize these influences by simultaneously measuring the concentrations of β-casein variants A1 and A2, as well as BCM-7, in raw milk and several widely used processed dairy products in a single experimental setup.

## 2. Results

**Figure 2:**
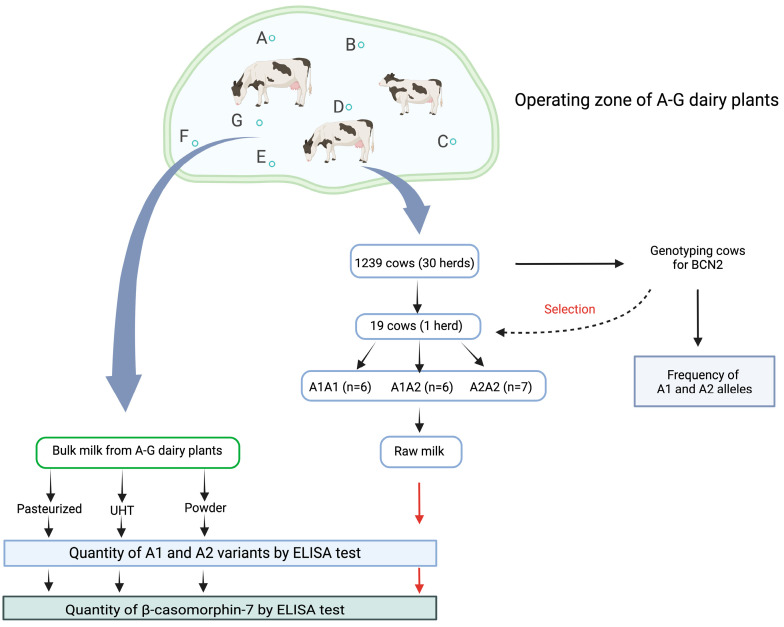
Workflow of experiments leading to measure the content of β-casein variants A1 and A2 as well as BCM-7 in the raw milk and popular processed dairy products. By A–G letters, 7 dairy plants are abbreviated.

The goal of our study was achieved in several experiments which are presented in the workflow scheme (Figure 2).

In an operating zone of seven dairy plants (A–G), 1239 randomly selected cows from 30 herds were genotyped for *CSN2 locus*, which allowed an estimation of the *CSN2* allele frequency for the entire cow population in the dairies’ farm zone. Out of the 1239 cows, 19 cows were selected from a herd with genotypes A1A1 (6), A1A2 (6), and A2A2 (7). Raw milk samples were taken from these cows to measure the amount of A1 and A2 variants and the amount of BCM-7 in the milk using the ELISA method. Three processed milk products (milk pasteurized, UHT milk, and milk powder) produced by dairies (A–G) were subjected to the same measurements of CSN2 variants and BCM-7 as raw milk.

### 2.1. Content of β-Casein A1 and A2 Variants in Dairy Products

**Figure 3:**
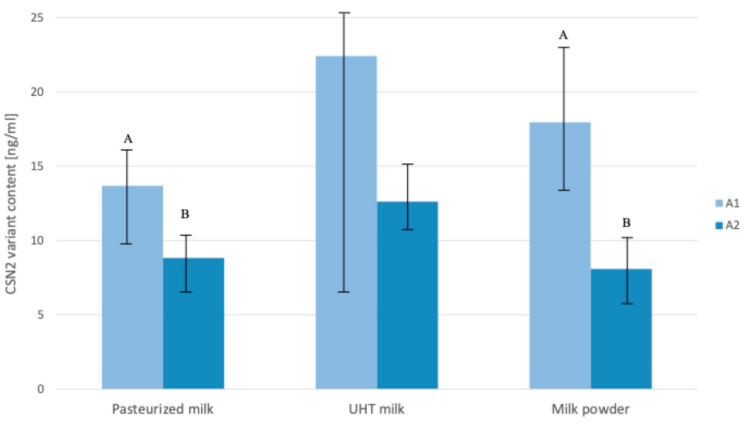
Relative content of β-casein A1 and A2 variants in raw milk and processed dairy products (pasteurized milk, UHT milk, and milk powder). Bars represent mean ± SD. Different superscript letters indicate significant differences (*p* < 0.01) among product types. Note: in UHT milk, difference between the content of A1 and A2 variants did not reach statistical significance (*p* > 0.05) due to high variability among samples. Different capital letters A and B indicate statistical significance between means at level *p* < 0.01.

The concentrations of β-casein A1 and A2 variants varied depending on the type of processed milk products (Figure 3). In pasteurized milk, the average level of the A1 variant was 13.69 ng/mL (SD = 5.38), which was significantly higher (*p* < 0.01) than that of the A2 variant—8.83 ng/mL (SD = 3.04). A similar pattern was observed for milk powder, where the A1 variant reached 17.95 ng/mL (SD = 8.02) while the A2 variant was present at a lower concentration of 8.10 ng/mL (SD = 4.66), which also represents a statistically significant difference (*p* < 0.01). In UHT milk, although the level of the A1 variant (22.41 ng/mL; SD = 15.24) was numerically higher than that of the A2 variant (12.62 ng/mL; SD = 6.07), the difference did not reach statistical significance, which was probably due to the high variability of the samples.

Very high R^2^ for A1 and A2 variant measurements (0.9916 and 0.9809, respectively) ensured the satisfied quality of beta-casein variants content estimation by ELISA in milk and milk products.

### 2.2. Content of β-Casomorphin-7 in Dairy Products

**Figure 4:**
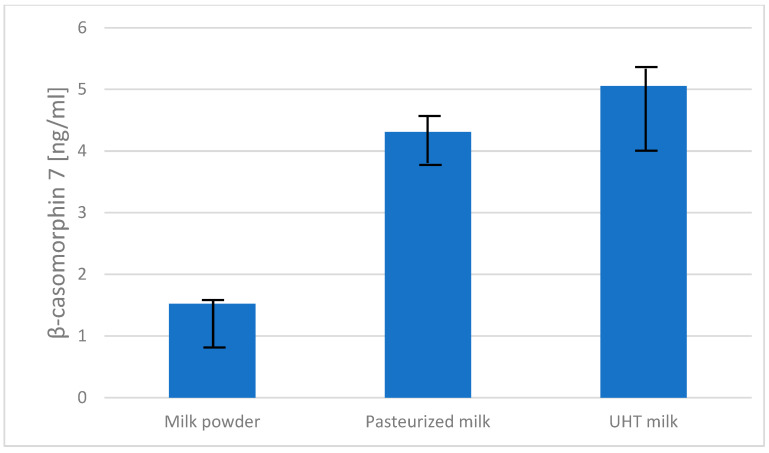
Concentration of β-casomorphin-7 in raw milk and processed dairy products (pasteurized milk, UHT milk, and milk powder), determined by ELISA. Bars represent mean ± SD.

The concentration of β-casomorphin-7 detected in the analyzed dairy products varied depending on the type of processing but showed no statistically significant differences between the product categories (*p* = 0.6426). ELISA R^2^ value was 0.9847. The highest mean of BCM-7 level was observed in UHT milk with 5.05 ng/mL (SD = 1.49), followed by pasteurized milk with a mean value of 4.31 ng/mL (SD = 0.94). The lowest concentration was found in milk powder at 1.52 ng/mL (SD = 0.88) (Figure 4).

### 2.3. Content of β-Casein Variants in Raw Milk

**Figure 5:**
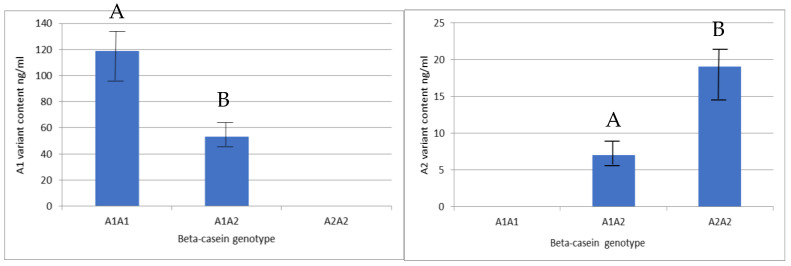
Mean values and standard deviation (± SD) of β-casein variant A1 (left) and variant A2 (right) contents in raw milk obtained from cows with identified *CSN2* genotype. Statistically significant differences (*p* < 0.01) are indicated with different superscript letters. Different capital letters A and B indicate statistical significance between means at level *p* < 0.01.

In order to provide a comparative value for the content of CSN2 variants in processed dairy products, the concentration of these variants was measured in raw milk (before processing), but in this case the milk samples were collected from cows with known *CSN2* genotypes (Figure 5).

The concentration of the A1 variant in the milk of cows with the A1A1 genotype was significantly higher (118.78 ng/mL, SD = 25.48) than in the milk of cows with the A1A2 genotype (53.40 ng/mL, SD = 6.55). In the milk obtained from cows with the A2A2 genotype, the content of variant A2 was significantly higher (19.07 ng/mL, SD = 3.08) than in the milk from cows with the A1A2 genotype (7.02 ng/mL, SD = 2.99) (Figure 5).

### 2.4. Content of β-Casomorphin-7 in Raw Milk

**Figure 6:**
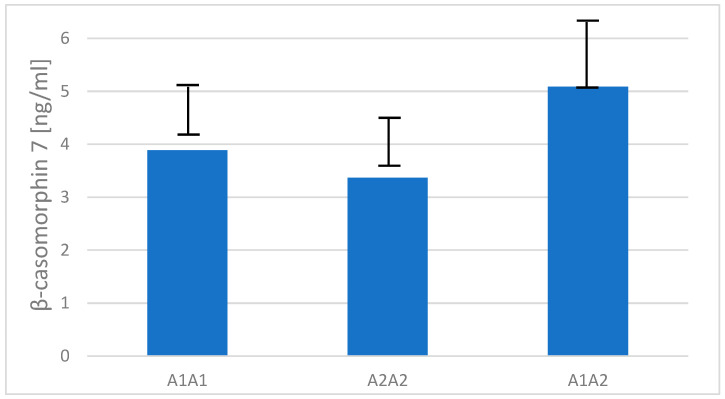
Mean values (± SD) of beta-casomorphin-7 in raw milk (before processing) collected from cows of different *CSN2* genotypes.

The same 19 samples of raw milk collected from cows with known β-casein genotypes were also used to detect variations in the amount of BCM-7. The results showed that milk from heterozygous A1A2 cows had the highest average BCM-7 content at 5.09 ng/mL (SD = 1.14), followed by A1A1 cows at 3.89 ng/mL (SD = 0.87). The lowest concentration of BCM-7 was found in the milk of homozygous A2A2 cows, with a mean value of 3.37 ng/mL (SD = 0.82), with no statistical differences (Figure 6).

### 2.5. Frequency of Beta-Casein Genotypes and Alleles in Holstein-Friesian Cows

**Figure 7:**
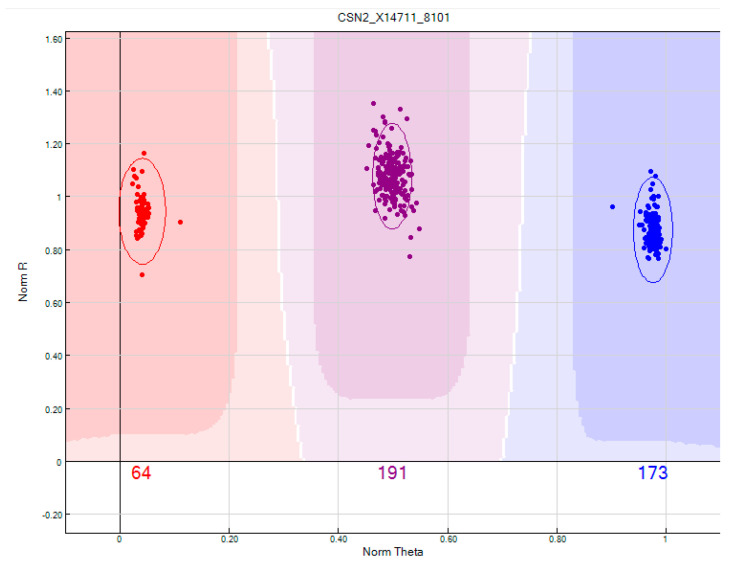
Cluster plot of SNP X14711:g.8101C>A genotyping (Illumina BovineSNP50 BeadChip). The *x*-axis shows normalized theta (genotype frequency), and the *y*-axis shows normalized R (signal intensity). Distinct clusters correspond to genotypes A1A1, A1A2, and A2A2, confirming high-quality SNP calling. In the figure, only part the whole population (1239 cows) is shown.

**Figure 8:**
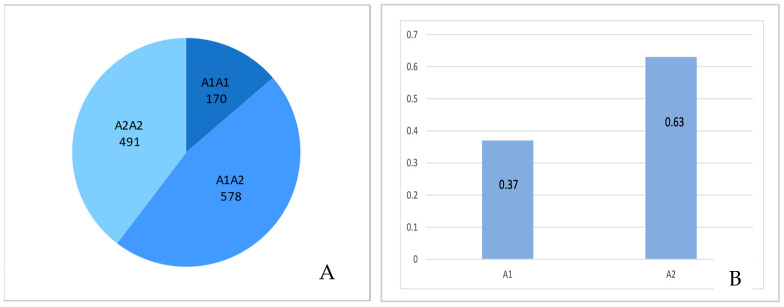
Frequency of *CSN2* genotypes (**A**) and alleles (**B**) in the sample of 1239 Holstein-Friesian cows milked in the operating zones of dairy plants included in the study.

Using the Illumina Bovine Chip, 1239 Holstein-Friesian cows were successfully genotyped. The overall call rate for the SNP *CSN2*_X14711_8101 (related to alleles A1 and A2 of *CSN2*) reached 100%. As shown in Figure 7, the quality of the genotype cluster was very good: dots representing the animals were distinctly separated into narrow clusters. From the whole population, 170, 578, and 491 cows with A1A1, A1A2, and A2A2 genotypes were identified, respectively. These numbers allowed us to calculate frequency of allele A1 and allele A2 as 0.37 and 0.63, respectively (Figure 8).

## 3. Discussion

Since the discovery of milk protein variants [26], it has been generally assumed that casein alleles follow a codominant pattern of inheritance in which each allele of a pair is expressed in approximately equal amounts. The first study to challenge this assumption was published by Ehrmann et al. [27], who investigated the relationship between milk protein genotypes and protein levels using densitometry of polyacrylamide gels. Their results showed a small but significant difference in the relative proportions of β-casein A1A1 and A2A2, expressed as a percentage of total milk protein. Unequal or monoallelic gene expression can be explained by at least three mechanisms: (1) variants within regulatory transcription factor (TF) binding sites that alter transcription rates of the affected allele; (2) differences in DNA methylation between maternally and paternally inherited β-casein alleles; and (3) allele-specific repression by microRNAs [28]. Evidence for regulatory polymorphisms affecting gene expression has also been reported for bovine β-lactoglobulin. For example, Davis et al. [29] identified a synonymous G/A SNP at +78 bp (exon 1) within the 5′UTR that caused an approximately 60% reduction in variant A mRNA expression compared to the wild-type G allele. However, the corresponding protein levels of β-lactoglobulin variants A and B in milk were not quantified. In addition, two SNPs were identified in the 3′UTR of β-casein, a region targeted by microRNAs, that may mediate allele-specific silencing [30].

The content of β-casein variants in bulk milk transported from farms to dairies and processed to obtain dairy products should reflect the frequency of β-casein alleles in the milked cow population. Since the frequency of the A1 variant in Holstein-Friesians was estimated at 37% and that of the A2 variant at 63% [Figure 8], it was expected that the amount of β-casein protein variants in bulk milk (a mixture of milk from many individual cows) should be similar, i.e., the content of the A2 variant should be higher than the A1 variant. However, in this paper, this theoretical assumption is verified and the inverse relationship is clearly shown—the quantity of the A1 variant is higher than the A2 variant in all three analyzed milk products (Figure 3). This result cannot be biased by a deviation in the beta-casein frequency (in favor of the A1 variant), as the bulk milk was a mixture of milk from 30 randomly selected farms (located in the operating zones of the dairies included in our study) in which 1239 cows with different genotypes of beta-casein were present. The current frequency of beta-casein alleles (Figure 8) was very close to the results previously obtained by our group for a larger population of Polish Holstein-Friesian cattle [23]. We believe that this unexpected result can be partly explained by our recent report [16] in which the phenomenon of unbalanced expression of *CSN2* alleles A1 and A2 was observed, possibly influenced by post-transcriptional or epigenetic regulatory mechanisms. In line with previous findings [16], we confirmed that in raw milk from cows with different β-casein variants, the A1 variant occurred at significantly higher levels than the A2 variant (Figure 5). The levels of β-casein variants observed in this study cannot be directly compared with the results reported by Duarte-Vazques et al. [31], where the mean relative abundance of A1 and A2 variants in raw milk of Holstein cows was 0.574 and 0.426, respectively. Since the milk analyzed in this study came from 40 Holstein cows with unknown β-casein genotype, it is assumed that both alleles were present, but the actual ratio of protein variants probably varied according to the proportion of A1A1, A1A2, and A2A2 genotypes within the group. In the same study, the A1:A2 ratio was also measured in four infant formula products. Although the differences were not statistically significant, the ratio showed greater variability depending on the type of formula. Furthermore, a direct comparison with the mass spectrometry results was not meaningful [32] as the ionization efficiencies of the different β-casein proteoforms were different. Therefore, the MS signal intensities for the A1 and A2 variants do not accurately reflect their actual mass ratios in the sample.

A higher level of variant A1 than A2 in processed milk should theoretically be reflected in a higher level of BCM-7, as this variant is the only source of this peptide.

In a report by Cieślińska et al. [33], the content of BCM-7 in the same types of processed milk (milk pasteurized, UHT, and milk powder) obtained from A1A1 and A1A2 milk was much higher than in A2A2 milk because the milk samples were treated with a cocktail of digestive enzymes, which is consistent with our current results showing higher content of the A1 variant in the same milk products (Figure 3). The low quantity of BCM-7 in milk products (Figure 4) and raw milk (Figure 6) is not surprising as the processed milk and raw milk samples were not digested by gastrointestinal enzymes.

Although BCM-7 can be released from both β-casein variants, its release from the A1 variant is much more pronounced than from the A2 variant. Only traces of BCM-7 were detected in undigested samples (i.e., raw milk and heat-treated milk). Our results confirm that these amounts are minimal and probably reflect the non-enzymatic peptide release by heat treatment, which alters the milk components and their interactions. Although the detected levels were low, the quantitative significance of these findings remains a matter of debate. However, after enzymatic digestion, the difference between the A1 and A2 variants becomes many times greater [34].

Although bulk milk is a complex of genetic and environmental factors, the overrepresentation of the A1 variant in the final dairy products was maintained and statistically confirmed for pasteurized milk and milk powder (Figure 3). For UHT milk, the differences were detected but not statistically confirmed, probably due to the high standard deviation of the samples. The fact that the dairy products were produced by seven different companies minimizes the differences in milk processing techniques used, although they could be the source of some variation, which probably occurred mainly in UHT milk.

The results for BCM-7 content (Figure 4 and Figure 6) showed no statistically significant differences between the milk products. However, UHT milk, which had the highest β-casein A1 content, also had the highest β-casomorphin-7 concentration. Although the differences were not statistically significant, the trend indicated that UHT milk had the highest average BCM-7 content (which may also be partly explained by heat treatment, promoting partial protein hydrolysis and non-enzymatic peptide release), followed by pasteurized milk, while powdered milk had the lowest one. These differences were small and insignificant, again possibly due to the fact that the milk samples were not digested by gastrointestinal enzymes capable of releasing BCM-7 from variant A1, as we described in a previous study [28] and also recently presented by Danesi et al. [25] who estimated daily BCM-7 exposure for adults, adolescents, and children. Traces of BCM-7 in dairy products could be a consequence of the self-digestion of casein, which is enhanced by the high-temperature treatment during milk processing [35].

BCM-7 concentrations were also measured in raw milk from cows with different *CSN2* genotypes (A1A1, A1A2, A2A2) (Figure 6). Milk from A1A2 cows had the highest BCM-7 concentrations, followed by A1A1, while A2A2 milk had the lowest concentration.

Although the absolute difference in BCM-7 concentration between A1A1 and A2A2 raw milk was only 0.52 ng/mL, this corresponds to a difference of about 13–15%. Although this difference is relatively small, it is not trivial, and its biological relevance may depend on individual sensitivity and clinical context. This clarification is important to avoid either over- or underestimating the potential impact of this variation.

This result supports the hypothesis that the presence of the A1 variant contributes to the formation of BCM-7 and that the A2 variant does not significantly produce this peptide, even in situations when β-casein protein is not undergoing proteolysis by digestive enzymes. These results suggest that the presence of the A1 variant is associated with increased BCM-7 release in raw milk, with the heterozygous A1A2 genotype possibly leading to synergistic effects in BCM-7 formation. Since BCM-7 levels were measured in milk that was not digested with proteolytic enzymes, it is expected that BCM-7 content would increase, as has been described in numerous publications [36,37,38,39].

It should be emphasized that the low concentrations of BCM-7 detected in undigested milk and dairy product samples (Figure 4 and Figure 6) cannot be directly extrapolated to human gastrointestinal exposure. β-casomorphin-7 is predominantly produced during gastrointestinal digestion as a result of enzymatic cleavage of β-casein, and its actual release in vivo depends on several factors, including proteolytic activity, peptide degradation, and individual variability in digestion. Therefore, our results only reflect the native presence of BCM-7 in milk and processed products prior to digestion and should not be interpreted in terms of potential health effects [40,41].

The method used for the identification of β-casein alleles, namely the Illumina Bovine Microarray, also allows for the detection of rare alleles such as A3, B, and I. However, in our study, individuals carrying these alleles were very rare and were therefore not included to maintain the integrity of the comparison between *CSN2* genotype and allele frequencies and the corresponding levels of β-casein variants in milk and dairy products. The reason for this is that the ELISA kit is only designed to detect two variants (A1 and A2), not all five. Furthermore, only these two variants (A1 and A2) differ in their ability to produce β-casomorphin-7; therefore, analyzing combinations of rare alleles would have resulted in very small groups of cows with limited statistical value. However, it should be recognized that in larger cow populations it is possible to identify a sufficient number of groups carrying rare genotypes such as A1I, A2I, A1B, and A2B, and thus extend the results obtained in our study, provided that an ELISA kit capable of detecting variants I and B become available.

The ELISA kit used in our study, which can only detect the two common β-casein variants in bulk milkme However, according to Santillo et al. [42], the A1 variant does not only represent milk exclusively from A1A1 cows, but also from rarer genotypes such as A1I or A1B. Therefore, milk in which only the A1 variant is detected should be labeled as A1-like milk. Similarly, milk in which only the A2 variant is detected, should be labeled as milk in which not only the A2 variant occurs (e.g., potentially A2A2, A2B, or A2I cows), as the milk may originate not only from A2A2 cows, but also from A2I or A2B cows. A similar approach was followed by Asledottir et al. [36], who categorized cow’s milk into two categories: the A1 family and the A2 family. Despite this diversity of variants and their combinations, A2 milk should only be obtained from cows with the A2A2 genotype from the consumer’s point of view in order to avoid contamination with other variants that reduce the positive effects of the A2 variant.

Different analytical methods with varying sensitivity and specificity are used to determine BCM-7. A commonly used screening method is the ELISA method, which is characterized by high sensitivity in the ng/mL range and allows a large number of samples to be analyzed in a short time. However, due to the risk of cross-reactions with other opioid peptides, quantitative results require careful interpretation and validation with instrumental methods [33,39]. In comparison to ELISA, HPLC with UV or fluorescence detection is a simple and well-established method, but it does not allow unambiguous identification of the peptide sequence [43]. The gold standard for the determination of BCM-7 is still LC-MS/MS, which ensures the highest specificity and allows the detection of trace amounts of BCM-7 in commercial milk and the quantitative monitoring of its release during in vitro digestion or in dairy products [36,44,45,46]. The ELISA test was chosen due to its availability, standardization, and the possibility of analyzing a large number of samples; it remains a useful tool for screening studies, while LC-MS/MS serves as a reference method for unambiguous identification and validation.

Since four casein loci form a tightly linked group, such that a change at one locus leads to changes at the remaining linked loci, it would provide additional insight into the reasons for the overexpression of the A1 variant compared to the A2 variant, as reported in this study and in our previous work [11]. When analyzing bulk milk—the form that reaches the consumer—we can reasonably assume that it contains an average combination of different genotypes across all milk protein genes (including the whey protein variants); therefore, the influence of other loci on the content of A1 and A2 β-casein variants is balanced by their random occurrence and for this reason can be largely neglected. Consideration of the influence of other casein loci on the expression of CSN2 variants would only be possible if commercial ELISA kits for variants of other milk proteins variants, such as κ-casein or αS-caseins, were available. The determination of all milk protein genotypes in individual milk samples would certainly make it possible to test the hypothesis that the genotypes of other milk proteins, e.g., κ-casein BB or AA, influence the content of A1 and A2 CSN2 variants as well as the composition and coagulation properties of milk, as suggested by Bonfatti et al. [11]. This represents a promising avenue of research that still needs to be explored, provided that a sufficiently large number of cows and milk samples are analyzed while controlling for other environmental factors that may influence the composition of milk and its properties. In this context, the β-casein B variant should also be considered, as it has been associated with favorable coagulation properties and cheese production performance. Although its frequency in Holstein-Friesian cattle in Poland is very low (2.7%; our unpublished data), future studies involving larger populations could help to clarify its contribution to milk processing traits.

The result of the current study is important for the social acceptance of the consumption of cow’s milk. From reports describing the frequency of β-casein alleles in Holstein-Friesian cattle over the last decade, it can be concluded that the undesirable A1 variant is less common [21,22,23] as bulls with A2 alleles are favored in breeding strategies. Our results suggest that this positive trend is not clearly visible in dairy products made from bulk milk, as the A1 variant is synthesized more efficiently than the A2 variant (Figure 5). This observation must be taken into account in any endeavor to reduce the content of variant A1 in conventional dairy products when approaching the production of A2 milk.

## 4. Materials and Methods

### 4.1. Genotyping of Beta-Casein Allele A1 and A2 in Cows

A population of 1239 cows of the Holstein-Friesian breed (black and white variety), randomly selected from 30 herds, were included in the study. The herds were located in the operating areas of the 7 dairies, which milk products were included in the study. Ear tissue samples were collected from the cows using Allflex technology (Allflex, Vitre, France) and used to isolate genomic DNA using the NucleoSpin Tissue Mini Kit (Macherey-Nagel GmbH, Berlin, Germany) according to the manufacturer’s protocols. The ear samples were collected as part of a routine veterinary procedure required for the evaluation of the genomic breeding value of the cows and therefore no authorization from the Bioethics Commission was required under Polish law. The genotypes of BCN2 alleles A1 and A2 were identified using the Illumina Bovine MDv2 Chip (Illumina Inc., San Diego, CA, USA). The quality of the SNP cluster was assessed using Illumina GenomeStudio software (v. 2011.1, Illumina Inc., San Diego, CA, USA). This variant is represented as marker *CSN2*_X14711_8101 and is able to distinguish between alleles A1 and A2.

### 4.2. Milk Samples

#### 4.2.1. Commercial Dairy Products

Three different types of milk products were included in the study: pasteurized milk, UHT milk, and milk powder. The products came from seven different dairy plants and were available in the usual grocery shops in Poland in a volume of 1 kg. These dairy plants operated in various regions, including 30 dairy farms from which DNA samples of cows were genotyped for *CSN2*.

#### 4.2.2. Raw Milk

Out of 1239 cows, 19 cows were selected with the A1A1 (*n* = 6), A2A2 (*n* = 7), and A1A2 (*n* = 6) *CSN2* genotypes. These cows were held in one herd, were in the first lactation, and showed very similar milk yield (ranged between 9800 and 10,500 kg) and milk total protein percentage (ranged between 3,39 and 3.44%). The cows showed no subclinical or clinical symptoms of udder inflammation; all cows were in good general condition. The cows were fed a total mixed ration (TMR) consisting of maize silage, grass and lucerne hay, and brewer’s grains, supplemented with a mineral and vitamin premix. The animals were housed in a free stall barn and were milked twice daily in a parallel milking parlor. Milk samples (1 L) were taken between 40 and 50 days of lactation from morning milking and immediately transported to the lab.

### 4.3. Measuring the Content of β-Casein A1 and A2 Variants by ELISA

After a brief homogenization, raw milk or dairy products were tested for the presence of A1 and A2 beta-casein variants using the Bovine A1 (or A2) Beta-Casein ELISA Kit (Biosensis Pty Ltd., Thebarton, Australia). This test utilizes a rabbit polyclonal anti-β-casein antibody pre-coated on microplate wells, a chicken-derived anti-β-casein antibody specific for the bovine A1 (or A2) variant, and a horseradish peroxidase (HRP)-conjugated donkey anti-chicken IgY antibody for detection. After the addition of the chromogenic substrate 3,3′,5,5′-tetramethylbenzidine (TMB), a colorimetric reaction occurs which produces a signal that is directly proportional to the concentration of A1 (or A2) β-casein in the tested sample or protein standard. The milk powder samples were first reconstituted with distilled water according to the manufacturer’s instructions. Then, 10 µL of each milk product was taken immediately after opening the package and diluted 1:40,000 in the assay diluent provided. An aliquot of 100 µL of each diluted sample was added to the wells of the pre-coated microplate. In parallel, A1 (or A2) β-casein standards and a blank (assay diluent only) were also added. The plate was sealed and incubated in an orbital shaker (OHAUS Corporation, New Jersey, USA) for 120 min. After incubation, the contents of the wells were discarded, and each well was washed five times with 200 µL of 1× wash buffer. This cycle of addition, incubation, discarding, and washing was repeated after successively adding 100 µL of the detection antibody, 100 µL of the donkey anti-chicken IgY-HRP conjugate, and finally 100 µL of the TMB substrate. The respective incubation times for these steps were 120, 60, and 20 min. The enzymatic reaction was terminated by adding 100 µL of the stop solution to each well. The absorbance was measured at 450 nm using a Multiscan FC Reader (Thermo Fisher Scientific, Waldham, MA, USA). All samples were analyzed in duplicate and the absorbance values were averaged. A standard curve was created by plotting the known β-casein concentrations on the *x*-axis and the corresponding optical density (OD) values on the *y*-axis. The OD values were converted to actual β-casein concentrations using MyAssays software v.11 (www.myassays.com) (MyAssays Limited, Brighton, UK)

### 4.4. β-Casomorphin-7 Extraction and Measurement by ELISA

The peptides were extracted from the mixture according to the method of Harwalkar and Elliott [47] with modification of Cieślińska et al. [48]. An amount of 200 mL of fresh milk was shaken for 1 h together with 200 mL of a 1:1 *v*/*v* chloroform/methanol mixture. The extract was made biphasic by adding 0.2 volume of distilled water. After 48 h, the lower layer was discarded and the upper layer containing peptides and amino acids was saturated with methanol (3:4 *v*/*v*). After 48 h, the mixture was centrifuged (3000× *g*/10 min) and the supernatant was evaporated (40 °C) and freeze-dried. The peptide extracts were purified by the SPE method using the STRATA C-18T, 140Ǻ, 50 μ column (Phenomenex, Inc., Torrance, California, USA).

β-Casomorphin-7 was detected using an enzyme-linked immunosorbent assay (Corning, Sigma-Aldrich, Poznan, Poland). The immunomodulator plates were first coated with specific antibodies and incubated at 37 °C for 2 h. Unbound antigens were removed by washing the wells with 200 µL of 1% phosphate-buffered saline with Tween 20 (PBS/Tween). To block non-specific binding sites, 200 µL of 1% gelatin was added and incubated at 37 °C for 1 h, followed by another PBS/Tween wash. Polyclonal antibodies were prepared according to the method described by Sienkiewicz-Szłapka et al. [43]. A volume of 100 µL of the antibody solution was added to each well, incubated for 1 h at 37 °C and washed with 200 µL of PBS/Tween. Subsequently, 100 µL of the secondary antibody (Sigma-Aldrich, Poznań, Poland) was added, followed by incubation at 37 °C for 1 h and a further wash with PBS/Tween. Subsequently, 100 µL of the peroxidase substrate o-phenylenediamine (OPD) was added and incubated at 37 °C for 30 min. The enzymatic reaction was stopped by adding 50 µL of 3 M hydrochloric acid (HCl). The absorbance was measured at 492 nm using an ELISA plate reader (ASYS UVM 340, Biochrome, Cambridge, England).

### 4.5. Statistical Analysis

Differences between the means of β-casein A1 or A2 variant concentrations and BCN-7 contents in raw milk and milk products were estimated by one-way ANOVA and a Duncan test by Statistica v 13 software package (StatSoft, Inc., Palo Alto, CA, USA).

## Data Availability

Detailed information is available upon request from the corresponding author. The raw data supporting the conclusions of this article will be made available by the authors on request.

## Abbreviations

The following abbreviations are used in this manuscript:

CSN2	bovine β-casein
BCM-7	β-casomorphin-7
SNP	single nucleotide polymorphism
